# Gibson approach and surgical hip dislocation according to Ganz in the treatment of femoral head fractures

**DOI:** 10.1186/s12891-021-04800-w

**Published:** 2021-11-17

**Authors:** Domenico De Mauro, Giuseppe Rovere, Amarildo Smakaj, Silvia Marino, Gianluca Ciolli, Andrea Perna, Concetto Battiato, Omar El Ezzo, Francesco Liuzza

**Affiliations:** 1 Department of Orthopaedics and Traumatology, Fondazione Policlinico Universitario A. Gemelli IRCCS, Largo Agostino Gemelli, 8, Rome, Italy; 2 Università Cattolica del Sacro Cuore, Rome, Italy; 3 ASUR Marche Area Vasta 5, “Mazzoni Hospital”, Ascoli Piceno, Italy

**Keywords:** Femoral head fractures, Pipkin’s fractures, Surgical hip dislocation, Ganz osteotomy

## Abstract

**Background:**

The purpose of the study is to evaluate clinical and radiological outcomes in those patients with femoral head fracture, treated with open reduction and internal fixation through Gibson approach and Ganz flip trochanter osteotomy. The treatment of Pipkin fractures is very challenging, especially for small trauma centers, because of the unusual fracture patterns and high-level surgical skills required.

**Case presentation:**

Between 2017 and 2020, nine cases of Pipkin fractures came to the Emergency Department at the Trauma Center of our Hospital in Rome. Inclusion criteria were the diagnosis of femoral head fracture, the open reduction and internal fixation as surgical choice and at least 24 months follow-up. Patients older than 65 years and those treated through total hip replacement or combined hip procedure (CHP) were excluded. Thus, five patients were included in our case series. The clinical outcome was evaluated according to Western Ontario and McMaster Universities Arthritis Index, Vail Hip score, modified Harris Hip score and Merle D’Aubignè Postel score. Radiographic assessment was scored according to Epstein-Thompson classification and heterotopic ossification was assessed through Brooker classification. The mean follow-up was 24 months (range 12-24). Average modified Harris Hip score was 92.1 points (range 75.9–100), and the average Vail score was 81.8 (range 55-95). WOMAC score was assessed in three different subscales, pain (A), stiffness (B) and physical condition (C), with the following results: 1.4 A (range 0-7), 1.2 B (range 0-6) and 6.4 C (range 0-22). Merle d’Aubignè Postel score resulted excellent for four patients and good for one patient. According to Epstein-Thompson score of the radiological outcome, four patients showed a good result and one a fair result. No mechanical or infective complications occurred in the five patients.

**Conclusions:**

Gibson’s approach and surgical hip dislocation through Ganz trochanteric flip osteotomy allow a good exposure of the femoral head and acetabulum, giving us the possibility to perform an anatomical reduction of the fracture. In our case series, satisfactory clinical and radiological short-term results were obtained without significant complications.

## Background

Femoral head fracture is a rare occurrence in the daily practice of a Trauma surgeon, often as a result of a high-energy trauma, such as road traffic accidents or falls from heights [1]. Nowadays the Orthopaedic Trauma Association and Arbeitsgemeinschaft für Osteosynthesefragen (AO) Foundation classifies these fractures as type 31C [2], but since 1957, the classification system introduced by Garrett Pipkin is commonly used [3]. Pipkin divided femoral head fractures in 4 categories: type 1 and type 2 are simple femoral head fractures, which are distinguished based on the involvement of the weight-bearing surface of the femoral head (type 2). In type 3, there is a concomitant femoral neck fracture, and in type 4 there is an associated acetabulum fracture, often the posterior wall due to the frequent association with posterior hip dislocation [4]. Diagnosis is based on X-rays as a first level diagnostic, but a CT-scan is needed to better understand the fracture and choose the best treatment option.

The treatment of Pipkin fractures is very challenging, especially for small trauma centers, because of the unusual fracture patterns and high-level surgical skills required [5]. There are different options for surgical treatment: small fragments excision in Pipkin I and total hip replacement in Pipkin III, open reduction and internal fixation (ORIF) of the femoral head fracture. Surgical dislocation of the hip is commonly required to reduce and fix the femoral head fragment. However, this procedure is associated with a high risk of damaging the ascending branch of the medial femoral circumflex artery, which is the most important blood supplier of the femoral head [6].

Several types of surgical approaches have been observed over the years: the anterior (Smith-Petersen, Hueter), antero-lateral (Watson-Jones) and posterior approach (Kocker-Langenbeck) are among the most used. We chose the modified Gibson approach as described by Moed in 2010 [7], using a straight skin incision. A more anterior interval is used, without violation of the gluteus maximus muscle fibers, preserving its neurovascular supply. Historically, treatment results have been rather poor, with high rates of early posttraumatic arthritis, osteonecrosis of the femoral head, heterotopic ossifications and sciatic nerve palsy.

Avascular necrosis of the femoral head is more frequent with the classical anterior or posterior approach, but the risk can be reduced using surgical hip dislocation through Ganz flip trochanter step osteotomy [8]. This surgical technique allows the simultaneous exposure of the acetabulum and the femoral head, without compromising the femoral head blood supply [9].

The aim of our retrospective cohort study was to describe clinical and radiological outcomes of a series of Pipkin fractures in our Department, treated by ORIF through surgical hip dislocation according to Ganz.

## Methods

### Patients

Between 2017 and 2019, nine cases of Pipkin fractures came to the Emergency Department at the Trauma Center of our Hospital in Rome. The year 2020 was excluded from the time interval, due to a progressive reduction of the emergency activities not COVID-19-related in our Hospital [10]. All fractures were caused by high-energy traffic accidents resulting in posterior hip dislocation. Hip dislocations were reduced in the emergency room in the first 6 h after the trauma, subsequently a distal femoral skeletal traction was positioned. Inclusion criteria were the diagnosis of femoral head fracture, the ORIF as surgical choice and at least 24 months follow-up. Patients older than 65 years and those treated through total hip replacement or combined hip procedure (CHP) were excluded. Average age was 39.8 years (range 17-53), four patients were male and one was a female. After following these inclusion criteria, five patients were included in the study, as they were treated by the same surgeon using ORIF with Gibson approach and surgical hip dislocation according to Ganz (Table 1).

**Table 1:** Patient characteristics, diagnosis and fracture classification

	Age	Sex	Diagnosis	AO	Pipkin
**1.**	33	F	sovrafoveal fracture + dislocation left femoral head	31C1.3	II
**2.**	49	M	sovrafoveal fracture left femoral head + posterior wall left acetabulum	31C1.3 + 62A1.2	IV
**3.**	17	M	sovrafoveal fracture + dislocation left femoral head	31C1.3	II
**4.**	53	M	sovrafoveal fracture right femoral head + dislocation right femoral head	31C1.3	II
**5.**	47	M	sovrafoveal fracture right femoral head + dislocation right femoral head	31C1.3	II

Femoral head fractures were diagnosed after complete radiographic investigation, including antero-posterior and Judet views. All patients underwent CT scan with multiplanar and 3D reconstructions to understand the fracture pattern and to accurately plan surgery.

**Fig. 1:**
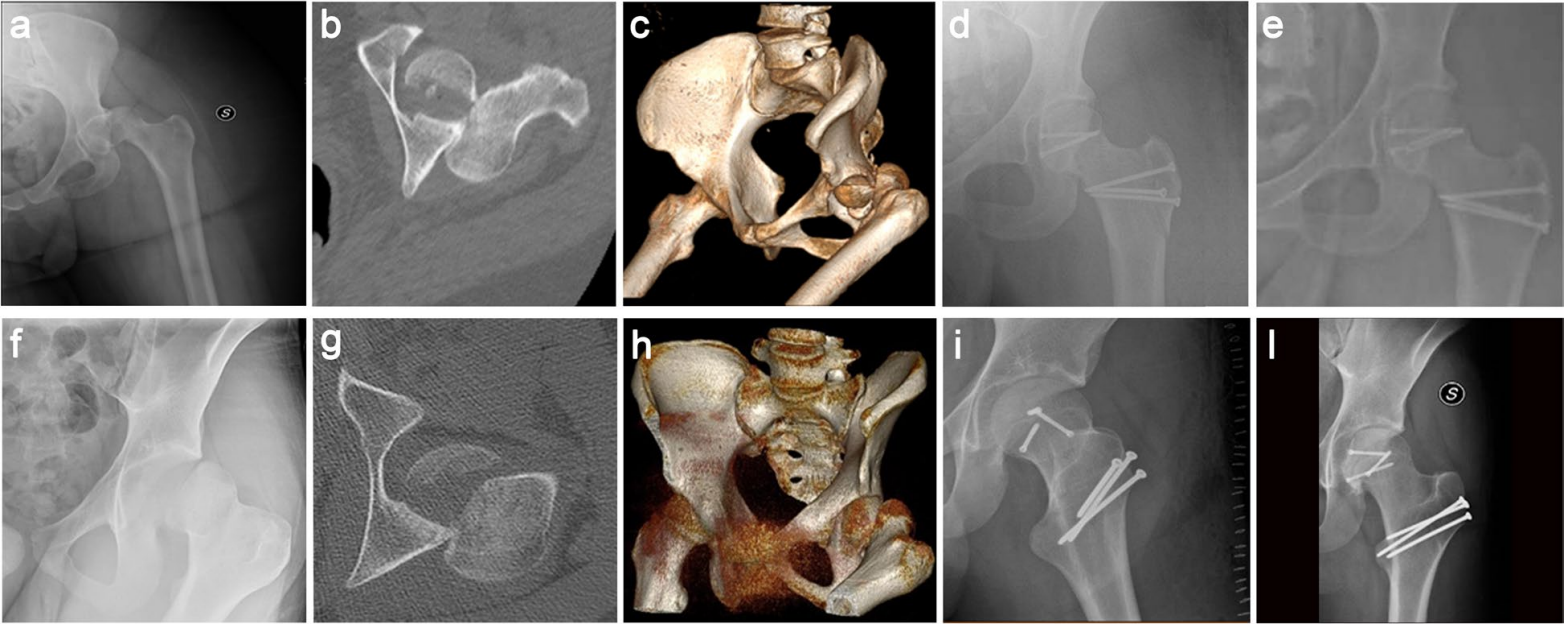
A 33-year-old woman (**a**-**e**) and a 17-year old man (**f**-**i**) with left femoral head Pipkin type II fracture treated by ORIF through Gibson approach and surgical dislocation with Ganz trochanteric flip osteotomy. The preoperative antero-posterior (AP) hip radiograph, axial CT and CT 3-D reconstruction after injury (**a**-**c**, **f**-**h**) and post-operative and final follow-up radiographs (**d**, **e**, **i**, **l**)

**Fig. 2:**
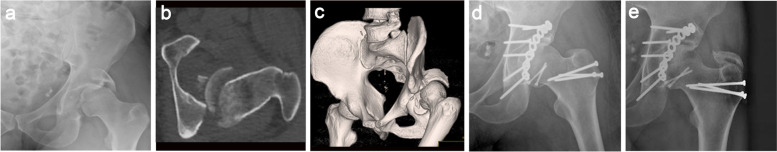
A 49 -year-old man (**a**-**e**) with a fracture of the left femoral head (Pipkin type IV) and of the posterior wall of the left acetabulum, treated by ORIF through Gibson approach and surgical hip dislocation with Ganz trochanteric flip osteotomy. The preoperative AP radiograph, axial CT and CT 3-D reconstruction after injury (**a**-**c**). Post-operative (**d**) and final follow-up radiographs (**e**)

According to Pipkin’s classification and AO-OTA classification, four fractures were classified as Pipkin II (31C1.3 AO Classification) (Fig. 1), one as Pipkin IV, due to a posterior wall fracture of the left acetabulum (31C1.3 + 62A1.2 AO Classification) (Fig. 2). The STROBE guidelines were used to ensure the reporting of this observational study [11].

### Surgical approach and implants

For patients with Pipkin II fracture, the modified Gibson approach was performed and the surgical dislocation of the hip was made through the trochanteric flip osteotomy as described by Ganz. The greater trochanter was osteotomized with a step osteotomy, as a variant of the technique originally described, to facilitate repositioning of the trochanter and its definitive fixation.

**Fig. 3:**
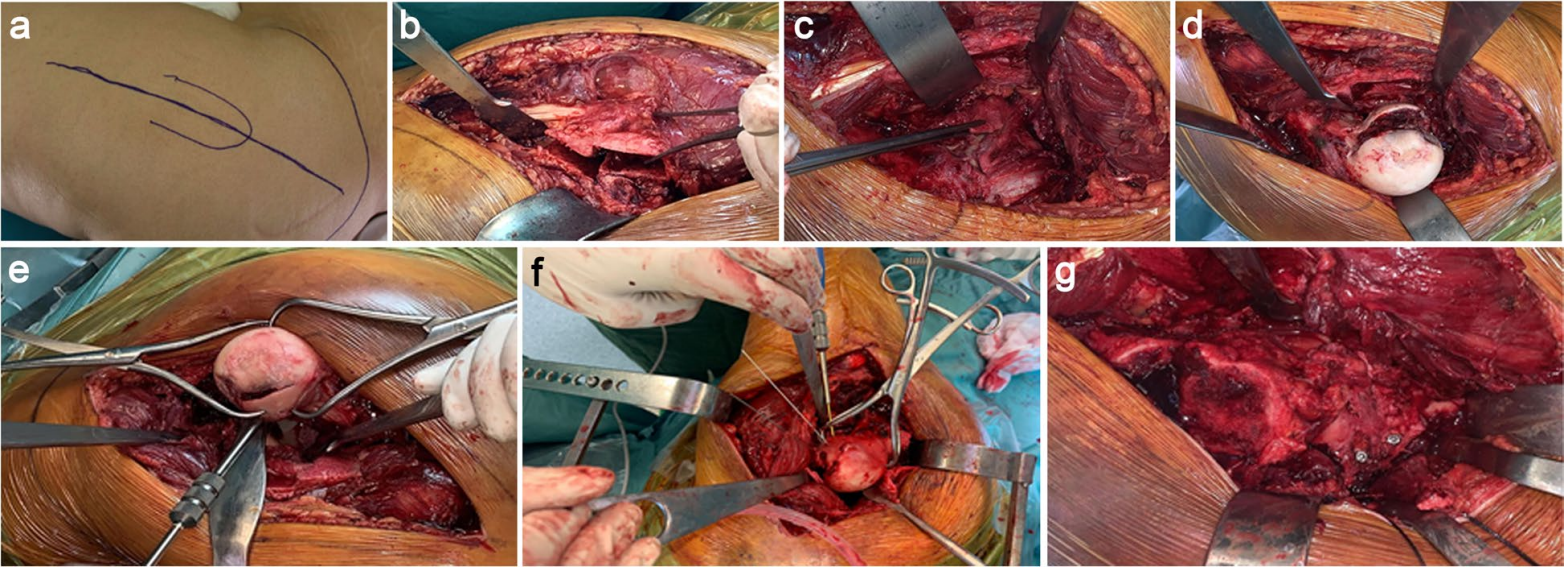
**a**-**g** Shows the steps of performing a safe surgical dislocation with a Ganz osteotomy for ORIF of a Pipkin type I and IV femoral head fracture: **a** Identified landmarks and planned incision. **b** Z shaped trochanteric osteotomy. **c** Z shaped Capsulotomy. **d** Exposed fracture of femoral head. **e** Fracture reduction with Weber clamps. **f** The fracture was reduced and then fixed with cannulated screws. **g** Reduction and synthesis of the greater trochanter

Fixation of the femoral head fragments was performed using three titanium 3 mm Herbert screws (HCS DePuy-Synthes). The greater trochanter was then repositioned and stabilized through three steel 3.5 mm screws (Fig. 3a-g). For the patient with a fracture of the femoral head and posterior wall of the left acetabulum (Pipkin IV), Gibson approach in lateral decubitus was chosen, and the fixation of the acetabular fracture was obtained through an 8-hole titanium plate with four screws, plus two free lag screws to obtain a more stable fixation of the posterior fragment (PRO Pelvis and Acetabulum System - Stryker).

### Post-operative protocol

An x-ray was performed the first day after the operation and all patients underwent the same post-operatory protocol. Thrombosis prophylaxis was achieved with low molecular weight heparin, elastic stockings and early bed mobilization. The patients were allowed to walk without weight bearing after 5 days. For 2 months, patients were treated with physical therapy with minimal weight bearing. Three months after surgery, full weight bearing was allowed.

Clinical and radiological follow-up was planned at first after 2 and 4 weeks, and was then repeated after 3, 6 and 12 months. After the first year, check up occurred every 12 months. Clinical outcome was evaluated according to Western Ontario and McMaster Universities Arthritis Index (WOMAC) [12], Vail Hip score (VHS) [13], modified Harris Hip score (mHHS) [14] and Merle D’Aubignè Postel score (MdA) [15]. These clinical scores consider the daily activities of the patients and their discomfort due to pain and functional limitations. WOMAC score has three different subscales: pain, stiffness and physical condition. The higher is the sum of points, the worse is the clinical outcome, with 0-20 range for Pain, 0-8 for Stiffness, and 0-68 for Physical Function. Vail hip score is a self-report scale of patient condition, including pain, daily activities limitation and gait, instead modified Harris Hip score takes into account also the range of motion of the hip. Both of them have 100 as maximum sum of points, but in mHHS the higher the score, the better is the outcome for the patient, as opposed to VHS. MdA score evaluates pain, gait and mobility of the patients, and a higher score in this test means a positive clinical outcome.

Radiographic assessment was made on plain anterior-posterior, obturator oblique and iliac oblique radiographs of the pelvis through Epstein-Thompson classification [16] and Brooker classification for heterotopic ossifications [17].

## Results

The mean follow-up was 24 months. Considering scores of the last follow-up visit average Harris modified hip score was 92.1 points (range 75.9–100), and the average Vail score was 81.8 (range 55-95). WOMAC score assessed through pain (A), stiffness (B) and physical condition (C), was: 1.4 A (range 0-7), 1.2 B (range 0-6) and 6.4 C (range 0-22). Merle d’Aubignè Postel score resulted excellent for four patients and good for one patient (Table 2). The complete range of motion was reached at the third month of follow-up. At the last follow-up, fractures were invisible on plain radiographs. According to Epstein-Thompson score of the radiological outcome, four patients showed a good result and one a fair result. No signs of post-traumatic osteoarthritis were observed during follow-up, compared to the contralateral side. No mechanical or infective complications occurred in the five patients. One patient developed heterotopic ossification after surgery (Brooker II) without clinical consequences.

**Table 2:** Results of the clinical scores at the last follow-up

No.	Last follow-up	VHS	MHHS	WOMAC	MdA
**1.**	36 months	95	100	A0,B0,C0	Good
**2.**	24 months	55	75.9	A7,B2,C22	Fair
**3.**	24 months	88	97.7	A0,B0,C0	Good
**4.**	24 months	83	90.2	A0,B2,C1	Good
**5.**	24 months	88	96.8	A0,B2,C9	Good

*WOMAC* Western Ontario and McMaster Universities Arthritis Index, *VHS* Vail Hip score, *MHHS* Modified Harris Hip score, *MdA* Marle d’Aubignè score

## Discussion

Femoral head fractures are commonly associated with high-energy trauma in young patients and in particular with dashboard injuries in car accidents. Thus, patients with Pipkin fractures often arrive in the emergency room as polytrauma. Posterior dislocations of the hip are associated to Pipkin fractures in 4 to 17% of cases [18] and prompt closed reduction in the emergency room is mandatory to prevent avascular necrosis of the femoral head [19]. Conservative treatment is an option for not-displaced fracture or small infrafoveal fragments, even if they are a rare occurrence. Complications associated with long immobility can occur in patients treated conservatively [20]. In order to decide which kind of surgical indication fits most, main factors such as fracture type, comorbidity and the age of the patient, should be considered. Usually, for young patients ORIF is the best option instead of fragment excision [21]. The latter is mostly performed when the fragment is small and there is no involvement of the bearing surface, such as Pipkin I. Instead, total hip arthroplasty is more suitable for elderly patients with an associated femoral neck fracture (Pipkin III), allowing them to start walking sooner, with lower complications rate. According to literature, Pipkin III fractures treated with Total hip replacement (THR) have a better outcome than those treated with ORIF, requiring a THR after failure of the internal fixation [22–24]. In elderly patients with fractures of both acetabulum and femoral head (Pipkin IV), fixation and total hip replacement represent a valid and definitive surgical option [25]. The most common surgical approaches for this type of fracture are two: anterior, Smith-Petersen/Hueter, and posterior, Kocher-Langenbeck (K-L). Neither of these approaches allows the surgeon a direct view and proper hip exposure [26]. In 2001, Ganz and his colleagues developed a different surgical technique [10], to allow a complete exposure of the hip and femoral head through digastric flip osteotomy of the greater trochanter. Since then, clinical trials evaluated this new approach showing positive results. Trikha et al. in 2018 [27] reported lower rates of complications in patients with acetabular or femoral head fractures treated through flip trochanter osteotomy, with good clinical outcomes. In his article, mean MdA score showed overall good to excellent result in 87.5% of cases. Lin et al. [28] reached similar results using the same technique, and the total rate of excellent and good outcomes was 77.3% in 22 patients with Pipkin I or II fractures, evaluated through MdA for clinical outcome and Thompson-Epstein for radiological outcome. Mostafa et al. in 2014 [29] had a higher rate of post-operative complications, especially HO and non-union of the osteotomy in the 11 patients with Pipkin I or II fractures treated according to Ganz technique, as compared with our group of patients. In this case the advantage of our study was the use of a “Z-shaped” osteotomy of great trochanter, which allowed a better and more stable reduction of the trochanter.

Even if femoral head fracture is a rare occurrence, our study reports a number of patients similar to case series already published, and therefore it further confirms and strengthens the positive conclusions recorded in Literature about flip trochanter osteotomy in the treatment of femoral head fractures.

As already said, these fractures are very rare, and even if our hospital is one of the largest trauma centers in Italy, the number of patients with a diagnosis of femoral head fracture is too small to provide statistical analysis to evaluate the effectiveness of the Ganz approach. The other limitation of our study is the lack of a control group of patients treated with a different surgical approach.

## Conclusions

Gibson’s approach and surgical hip dislocation through Ganz trochanteric flip osteotomy allow a good exposure of the femoral head and acetabulum, giving us the possibility to perform an anatomical reduction of the fracture. Our preliminary experience with this surgical technique, for femoral head fractures, seems to be encouraging. In our case series, satisfactory clinical and radiological short-term results were obtained without significant complications. Further and larger studies are needed to better understand the best treatment option for Pipkin fractures, with a longer follow-up for a better evaluation of clinical and radiological outcomes.

## Data Availability

The datasets used and/or analyzed during the current study are available from the corresponding author on reasonable request.

## Abbreviations

ORIF: Open reduction and internal fixation
AO: Arbeitsgemeinschaft für Osteosynthesefragen
CT: Computed tomography
CHP: Combined hip procedure
THR: Total hip replacement
WOMAC: Western Ontario and McMaster Universities Arthritis Index
VHS: Vail Hip score
mHHS: Modified Harris Hip score
MdA: Merle d’Aubignè Postel score
K-L: Kocher-Langenbeck
